# Safe Drugs Versus Innovative Drugs (Can We Have Both?)

**DOI:** 10.34172/apb.2020.041

**Published:** 2019-12-14

**Authors:** Vera Lúcia Raposo

**Affiliations:** Faculty of Law, Macao University, Macao, China.

Drugs are especially dangerous products and because of this a very demanding evaluation process is imposed on them before they reach the market. Due to previous episodes in which severe injuries were caused by drugs, such as in the Thalidomide case, nowadays in most legal systems drugs can only enter into the market after a rigorous approval process. At the end of this, a drug may obtain a marketing authorisation (MA) and can then be commercialised and used by patients, although there are some exceptions.

However, the demanding nature of the drug approval procedure raises some issues. Drugs are essential for promoting and maintaining health, improving quality of life and even avoiding death. Accordingly, if the approval process is too long or too demanding, the chances are that the drug will arrive on the market too late for some patients or will not arrive at all, either because the MA has been denied or because the pharmaceutical company abandons it, overwhelmed by its many legal requirements and liabilities and by the huge investment involved.

The drug approval procedure involves a very difficult balance. The approval process must be demanding enough so that only the safest drugs are selected, but it cannot be so demanding that patients do not have access to new medicines that are essential to their life and well-being.

Unlike other products, in the case of drugs safety cannot be defined as the total absence of risk, because in this particular context that goal is impossible to achieve. Instead, a drug is considered safe when its risks are tolerable, based on an analysis of its expected benefits and the existing therapeutic alternatives.^1^ In other words, a drug is never totally safe, but merely safe enough.

One of the most well-known studies on the dangers of drugs involved Thalidomide,^2^ an active substance commercialised by the pharmaceutical company Grunenthal in the 1950s under the trade name of Contergan. Although this drug was created as an antidote to poisonous gas, it was launched on the market in 1957 to combat morning sickness associated with pregnancy. At the time, there were no drug evaluation procedures, so the product was marketed without conducting specific studies or undergoing further evaluation. Rapidly it was commercialised all over Europe and even in other parts of the world (although it was never authorised in the United States) and it became very popular with pregnant women. However, after a few years, the medical community found a connection between taking this medicine and the birth of children with several congenital deformities, especially phocomelia, i.e., the absence of limbs. Eventually, the drug was withdrawn from markets around the world; however, between 1957 and 1961 (when the drug was withdrawn) more than 10 000 children in 46 different countries were born with serious anomalies. This sad episode shows how the demanding process of drug approval is essential to guarantee safety and efficiency.

Unfortunately, incidents with defective drugs do not belong to the past. In recent times there have been reports of people experiencing severe adverse drug reactions (ADRs) to products whose detrimental side effects were undetected (or undisclosed) by pharmaceutical companies in time. For instance, some years ago the blockbuster drug Vioxx was marketed by the pharmaceutical company Merck, which claimed the drug could treat pretty much everything from arthritis pain to menstrual cramps. However, when several patients suffered heart attacks, the medical community realised something was wrong and in 2004 the medicine was finally withdrawn (it was the biggest drug recall until that moment, involving products valued at $27 billion). According to a study from 2004, performed by Dr David J. Graham with Kaiser Permanente, the Vioxx painkiller might have contributed to 27 785 heart attacks and deaths between 1999 and 2003. The study concluded that people taking Vioxx were more likely to have heart attacks or die from sudden cardiac arrest than people taking a competing painkiller.^3^ However, the most striking part of this story was not the recall, but the suspicion that the pharmaceutical company might have been aware of the risk long before the first incidents. Whereas Merck argued that it was completely oblivious to the risk, a study published in 2006 in the prestigious *New England Journal of Medicine* stated differently, accusing Merck of having data showing the risk of cardiovascular episodes substantially before their occurrence.^4^ One curious fact is that in 2007 Merck agreed to pay $4.85 billion in compensation to victims of Vioxx; however, according to the *New York Times*, this amount represented only nine months of Merck’s profits from Vioxx sales. This value is especially impressive considering that the product was on the market (and profitable) for five years.^5^

Because there have been so many cases with ADRs, and especially the Thalidomide case, a rigorous drug approval procedure has been implemented in most jurisdictions. The procedure involves several years of research and clinical trials, and in the end all of the material is submitted to a competent drug authority that grants (or not) the MA, without which the drug cannot be used (although with some exceptions). If the drug passes all these phases, it is then evaluated by a competent drug authority that decides whether the drug can be marketed based on the results of the clinical trials submitted by the pharmaceutical company.

The drug approval procedure is often criticised for its excessive rigidity and level of demand. Yet it exhibits some weaknesses, and indeed many drugs arriving on the market end up showing ADRs years later.^6^ Both in Europe and the United States, serious ADRs have been detected in about 10% of the drugs that have been allowed into the market.^6^

One of the main weaknesses of the existing drug approval procedure relates to clinical trials and their limitations^7^:

i) Participants in clinical trials are carefully chosen, so they can hardly serve as models for the population to which the drug will be prescribed. For example, vulnerable populations like pregnant women, the elderly and children are not usually called for clinical trials, which means that there are virtually no authorised medicinal products for these groups. However, in practice drugs are often used on all these patients, without clinical trials supporting such use. Thus, the consequences are often unknown, and some may be potentially disastrous. Poly-medicated patients are also excluded, so it is impossible to draw conclusions about possible drug interactions;
ii) Clinical trials are temporarily limited, so they do not allow the detection of ADRs that are only identifiable in long-term treatments;
iii) Clinical trials monitor the drug when taken under optimal conditions, leaving out cases of abuse, misuse, forgetfulness in taking the medicine and other errors, which in everyday life turn out to be very frequent among patients;
iv) Clinical trials are intended more to control the effectiveness of the drug than its safety, and they rarely provide information on ADRs or on drug’s toxicity.

How can these limitations be annulled? Certainly, the already complex, time consuming and demanding process of drug approval can be further strengthened. However, the existing model is already too heavy and even limitative of R&D, so making it even more demanding would only hamper the arrival of innovative drugs on the market. Clinical trials are the first major barrier, and according to studies conducted on this issue 95% of all drugs that start being developed never reach the market, especially if they fail in clinical trials.^8^ It may also be that a drug does not obtain the necessary MA or that although it is authorised for commercialisation it is withdrawn from the market due to ADRs. Thus, pharmaceutical companies may invest millions and spend more than a decade focused on a medicinal product that in the end fails. When that happens, besides having no return at all or not enough return, the pharmaceutical company may still struggle with litigation and compensation. These contingencies turn the development of medicines into a risky business.

The problem is not that pharmaceutical companies are making less profit, because the pharma business is still one of the most lucrative ones. The problem is that if pharmaceutical companies do not have the expected profit they will stop investing in R&D, and without investigation twenty years from now we will still be using the good old aspiring to treat the new virus that each day come around.

Moreover, the strong demands of this procedure have given rise to some important consequences.^2^ On the one hand, it has generated within the community the (utopian) claim that duly approved drugs are totally safe, which is unfounded. On the other hand, the process of creating new medicines has been restricted to the largest pharmaceutical companies because only they can pay for the extensive R&D required and incur the inherent financial risk. This has substantially delayed the appearance of new products on the market, causing serious harm to patients in need.

As already mentioned, the MA does not provide an absolute guarantee of the safety and efficacy of a product.^9^ Absolute safety is actually an impossible goal to achieve, no matter how many studies and clinical trials are performed. Therefore, *prima facie,* there is no legal basis for suing the drug regulatory authority for granting an MA to a product that in the end causes severe harm to a patient, unless it is established that the authority did not perform its duties as stipulated under the law.^10^ For example, if an MA is issued after a negative risk-benefit analysis and the regulatory entity does not refuse to grant the MA, legal responsibility will certainly be imposed on the drug regulatory authority based on negligence. The same applies when the regulatory authority does not detect a risk that, although not contained in the dossier submitted by the pharmaceutical company in its MA application, should have been identified if the authority had performed its duties following the proper standard of care. Besides these exceptional circumstance the drug approval entity cannot be held liable for the injuries caused by the approved drug.

The best way to control safety hazards is to invest in pharmacovigilance. The term pharmacovigilance refers to the obligation to report ADRs after a medical product has entered the market. According to the WHO, ‘[p]harmacovigilance (PV) is defined as the science and activities relating to the detection, assessment, understanding and prevention of adverse effects or any other drug-related problem’.^11^ In fact, however comprehensive clinical trials may be, it is impossible to identify all of a product’s risks, which sometimes can only be detected after several years of being on the market.

Pharmacovigilance aims to identify new ADRs as soon as possible; to enrich existing information on previously identified ADRs; to compare the benefits of medicinal products with other existing medicines and to disseminate the conclusions obtained with the purpose of improving clinical practice.^7^ ADRs are estimated to account for 5% of hospitalisations within the European Union (EU), resulting in 197 000 deaths per year and a cost of some 79 billion euros.^12^

The occurrence of ADRs is not geographically uniform because it is conditional on several factors: the predominant population pathologies, the genetics of the national human group, the prevalent diet, the medications available, the tendencies in prescribing drugs (dosage, posology), the interconnection between drugs that are typical of Western medicine and traditional medicine products. These factors explain why the results obtained in a clinical trial held in one region cannot be blindly transposed to another.^7^

A risk-benefit analysis is first carried out at the time the MA is granted, but it may be repeated if, after commercialisation, serious ADRs are identified. The results of pharmacovigilance may change the risk-benefit assessment originally made in granting an MA, and thus lead to the revocation, suspension or modification of the original authorization.

For example, veralipride was authorised in several European member states, until it was withdrawn from the Spanish market in 2006 due to reports of serious ADRs on the patients’ nervous system. The European Medicines Agency was called upon to rule on the matter and finally recommended the withdrawal of all medicinal products containing veralipride because it considered the risk/benefit analysis performed during the marketing phase to be negative.^13^

The high level of demand that guides the risk-benefit assessment mechanism may have benefits, but it also has drawbacks. For instance, it may force pharmaceutical companies to withdraw products that, while not absolutely safe, are of great benefit to patients in need, and whose very existence depends on those medicines.

Let’s take the case of the vaccine against rotavirus. In 1998 the pharmaceutical company Wyeth (now Pfizer) introduced an oral vaccine against rotavirus, a virus that causes diarrhoea, and that in certain areas of the globe, such as sub-Saharan Africa, has been a leading cause of death in early childhood. Although the drug was quite effective in combating rotavirus, it was found to be associated with a higher incidence of intussusception, a condition in which one segment of the intestine bends within another segment, but rarely with lethal consequences. Even though it was not a serious ADR and its incidence was not widespread (it was estimated that 1 case per 10 000 vaccinated children), Wyeth chose to withdraw the product from the market, possibly fearing endless litigation and awards of heavy compensation. However, the risk-benefit assessment that was valid for the West did not apply to certain areas of Africa, where the absence of this vaccine became associated with high infant mortality. The drug was withdrawn in 1999, but another vaccine of the same type did not appear until 2006. During this interregnum period about three million African children died of rotavirus infection. Certainly, not all cases could have been avoided by using Wyeth’s vaccine, but many would have been.^2^ This example demonstrates how tricky risk assessment can be and shows that sometimes the community must accept a minor risk to achieve a higher benefit. If a proper risk-benefit assessment has been conducted, some drugs falling into this category must be authorised to enter the market because the benefits surpass the risks.

In sum, the existing drug approval process is already complicated, time-consuming and expensive, so it cannot become even more demanding; otherwise patients will not have access to new drugs. Although it is true that pharmaceutical companies make huge profits with this business, they also run huge risks because for some investments there is simply no return and with no profit there will not be more R&D, thus, there will not be innovative drugs. Accordingly, the approval procedure must remain demanding, but not too demanding.

Pharmacovigilance is the best solution for this impasse. We must implement an efficient and speedy mechanism for the post-marketing surveillance of ADRs, thus allowing new drugs into the market without imposing excessive obstacles, but at the same time be alert for potential ADRs. The aim is to withdraw drugs whenever the risk-benefit assessment becomes negative.

On the other hand, patients must realise that no drug is completely safe and that whenever they take a drug they are accepting the risk that accompanies it (which, in turn, assumes that the patient has been previously informed of the most relevant risks) in exchange for the benefits it provides.

In the case of non-pharmaceutical products, only the safest should ever be allowed to enter the market. But when it comes to drugs we must accept that there are always side effects, some of which potentially very dangerous.

## Ethical Issues

Not applicable.

## Conflict of Interest

Authors declare no conflict of interest in this study.

## Acknowledgments

This work was supported by the University of Macau Multi-Year Research Grant MYRG2015-00008-FLL.
